# Impact of COVID-19 vaccine on epilepsy in adult subjects: an Italian multicentric experience

**DOI:** 10.1007/s10072-022-06100-0

**Published:** 2022-05-02

**Authors:** Marina Romozzi, Eleonora Rollo, Paolo Quintieri, Fedele Dono, Giacomo Evangelista, Stefano Consoli, Luigi Veleno, Francesca Anzellotti, Carmen Calvello, Cinzia Costa, Serenella Servidei, Paolo Calabresi, Catello Vollono

**Affiliations:** 1 Dipartimento Universitario Di Neuroscienze, Università Cattolica del Sacro Cuore, Rome, Italy; 2 Neurologia, Dipartimento Di Scienze Dell’invecchiamento, Neurologiche, Ortopediche E Della Testa-Collo, Fondazione Policlinico Universitario Agostino Gemelli IRCCS, Rome, Italy; 3 Dipartimento Di Neuroscienze, Imaging E Scienze Cliniche, “G. D’Annunzio, Università Di Chieti-Pescara, Chieti, Italy; 4 Behavioral Neurology and Molecular Neurology Units, Center for Advanced Studies and Technology - CAST, University G. d’Annunzio of Chieti-Pescara, Chieti, Italy; 5 Sezione Di Neurologia, Dipartimento Di Medicina E Chirurgia, Università Di Perugia, Ospedale S. Maria della Misericordia, Perugia, Italy; 6 Neurofisiopatologia, Dipartimento Di Scienze Dell’invecchiamento, Neurologiche, Ortopediche E Della Testa-Collo, Fondazione Policlinico Universitario Agostino Gemelli IRCCS, Rome, Italy

**Keywords:** COVID-19, Coronavirus, Epilepsy, Vaccine, Seizures

## Abstract

**Objectives:**

To investigate the safety and tolerability of COVID-19 vaccines in people with epilepsy (PwE).

**Methods:**

In this multicentric observational cohort study, we recruited adult patients (age > 18 years old) with epilepsy who attended the Outpatient Epilepsy Clinic from 1st July to 30th October 2021. We administered to the patients a structured questionnaire and interview on demographic and epilepsy characteristics, current treatment, previous SARS-CoV-2 infection, vaccine characteristics, post-vaccine seizure relapse, other side effect, variation of sleep habits, caffeine, or alcohol intake. Seizure frequency worsening was defined as a ratio between mean monthly frequency post-vaccination and mean monthly frequency pre-vaccination superior to 1. Patients were categorized in two groups: patients with seizure frequency worsening (WORSE) and patients with seizure stability (STABLE).

**Results:**

A total of 358 people participated with a mean age of 47.46 ± 19.04. Focal seizure (79.1%), generalized epilepsy (20.4%), and unknown types of epilepsy (0.5%) were detected among participants. In total, 31 (8.7%) people expressed that they were not willing to receive a COVID-19 vaccine; 302 patients (92.35%) did not experience an increase in the seizure frequency (STABLE-group) whereas 25 patients (7.65%) had a seizure worsening (WORSE-group). Post-vaccine seizures occurred mainly in the 7 days following the administration of the vaccine. Patients in the WORSE-group were treated with a mean higher number of anti-seizure medication (ASMs) (*p* = 0.003) and had a higher pre-vaccine seizure frequency (*p* = 0.009) compared with patients in the STABLE-group. Drug-resistant epilepsy was also associated with seizure worsening (*p* = 0.01). One-year pre-vaccination seizure frequency pattern demonstrated that patients in the WORSE-group had a higher frequency pattern (*p* < 0.001). Multivariate analysis of the vaccinated group showed that only the seizure frequency pattern (confidence interval [CI] = 1.257–2.028; *p* < 0.001) was significantly associated with seizure worsening.

**Conclusion:**

In our cohort of vaccinated PwE, only a little percentage had a transient short-term increase of seizure frequency. The present study demonstrates that COVID-19 vaccines have a good safety and tolerability profile in the short term in PwE.

## Introduction

Coronavirus disease 2019 (COVID-19), caused by acute respiratory syndrome coronavirus 2 (SARS-CoV-2), has become a global pandemic [1]. Several vaccines for COVID-19 are now available and represent the most effective intervention to radically reduce the incidence of severe disease and death caused by SARS-CoV-2 infection. To date, 194 vaccines against COVID-19 are in preclinical evaluation and 132 vaccines are in clinical development, while 12 vaccines were approved by regulatory authorities [2]. During the study time, Pfizer/BNT162b2, Moderna Vaccine/mRNA1273, AstraZeneca/AZD122/ChAdOx1 n-CoV-19, and the Janssen vaccines/Ad26 were approved in Italy [2].

Up to now, the approved COVID-19 vaccines have demonstrated in clinical trials to be effective and safe [3–5].

According to literature, vaccines have been sporadically associated with neurologic complications including the occurrence of afebrile and febrile seizures, in particular in pediatric population [6, 7]. Seizure-relapse risk can be higher in patients with post-vaccination fever, a known factor which can lead to temporarily lower seizure threshold [8]. The importance of an effective vaccination campaign in people with epilepsy (PwE) lies behind the higher mortality and morbidity risk which these patients may have [9]. This issue is particularly relevant in the context of SARS-CoV-2 infection and COVID-19 where it has been shown that people with active epilepsy present a higher COVID-19 cumulative incidence and higher risk of mortality compared to general population [9].

Based on clinical trials, the *International League Against Epilepsy* (ILAE) recommended that PwE should receive COVID-19 vaccine (https://www.ilae.org/patient-care/covid-19-and-epilepsy/covid-19-vaccines-and-people-with-epilepsy). Moreover, PwE, as a vulnerable category, were listed as a priority group in Italy as other European countries during COVID-19 vaccination campaign (https://www.salute.gov.it).

However, real-world studies on PwE and safety profile of COVID-19 vaccination are underrepresented in the current literature.

This study aims to examine the safety and tolerability of vaccinations against COVID-19 in PwE and their correlation with epilepsy features.

## Methods

### Setting and participants

The study is a multicentric observational retrospective cohort study conducted across the COVID-19 vaccination campaign. We consecutively recruited patients with a diagnosis of epilepsy according to the International League Against Epilepsy (ILAE) criteria who were admitted to the Outpatient Clinics from three Italian tertiary referral epilepsy centers (Fondazione Policlinico Universitario A. Gemelli IRCCS, Rome; “G. d’Annunzio,” University of Chieti-Pescara, Chieti; University of Perugia, Ospedale S. Maria della Misericordia, Perugia) between 1st July and 30th October 2021.

Four vaccines were approved in Italy at the time of the study: Pfizer/BNT162b2, Moderna/mRNA1273, AstraZeneca/AZD122/ChAdOx1 n-CoV-19, and the Janssen/Ad26.

We included in the study patients > 18 years old, who gave informed consent to participate and had received two doses of vaccines, or who had received a single dose of vaccination because of a previous SARS-CoV-2 documented infection and in case of vaccination with Janssen vaccines/Ad26, and evaluated seizure frequency until 90 days from the first dose. Patients with mild cognitive impairment or mild-to-moderate intellectual disability were included in the study only if a caregiver was available for providing accurate information about the patient. Exclusion criteria were refusal to give informed consent and not fluent Italian speakers.

We also collected data on PwE who were not vaccinated at the time of the visit and their reasons for not being vaccinated.

### Data acquisition

Clinical data on epilepsy and COVID-19 vaccinations were obtained via a structured questionnaire and interview. The questionnaire comprised the following information: demographic (age, sex, ethnicity), epilepsy characteristics (seizure and epilepsy type, etiology, age of disease onset and duration, 1-year pre-vaccination seizure frequency pattern, 90 days pre-vaccination seizure frequency), current treatment, including anti-seizure medication (ASMs), ASMs variation in the previous year, other concomitant treatments, previous SARS-CoV-2 infection, type of vaccine and date of vaccination, post-vaccine seizure relapse (occurrence and timing of seizures after the scheduled vaccine doses, type of seizure and need for hospitalization due to seizure relapse), other side-effect due to vaccination (local or systemic reactions), variation of sleep habits, caffeine or alcohol intake.

Regarding sleep habits, we calculated the sleep efficiency that is the percentage of time spent asleep while in bed, by dividing the amount of time spent asleep (in minutes) by the total amount of time in bed (in minutes). A normal sleep efficiency is 85% or higher [10].

The questionnaire was administered by trained Neurologists, specialized in epilepsy.

Furthermore, the Beck Depression Inventory (BDI) [11] was administered to evaluate the presence of key symptoms of depression.

Seizures and epilepsy were classified according to the ILAE for both seizure type, epilepsy type, and etiology [12]. Drug-resistant epilepsy was also defined according to ILAE guidelines [12]. For each patient, seizure frequency in the 1-year pre-vaccination period was reviewed on the basis of patient’s self-diary and medical files and categorized in five sub-groups (< 1 seizure/year, > 1 seizure/year, > 1 seizure/month, > 1 seizure/week, > 1 seizure/day). Mean monthly frequency was assessed in the 90 days before vaccination and in the 90 days after the first dose of vaccine.

Seizure frequency worsening was defined as a ratio between mean monthly frequency post-vaccination and mean monthly frequency pre-vaccination superior than 1.

Hence, patients were categorized in two groups: patients with seizure frequency worsening (WORSE) and patients with seizure stability (STABLE, i.e., patients with no variation in seizure frequency or patients with decreased seizure frequency).

Non-vaccinated patients were asked to report their concerns about COVID-19 vaccination.

### Statistical analysis

Statistical analysis was performed in multiple steps. Descriptive statistics were used to describe demographic and clinical features of the sample. Numerical variables were described using the following measures: mean and standard deviation. Categorical variables were presented as absolute number (*n*) and percentage.

After testing all numerical variables for normal distribution, univariate analysis was performed by means of the Shapiro–Wilk test.

In order to compare numerical variables, we used a nonparametric test (Mann–Whitney *U*-test); for categorical variables, we adopted the Pearson’s chi-square (*χ*^2^). The level of significance was set at *p* < 0.05.

In a further step, variables compared in the univariate analysis were entered into a multivariate logistic regression analysis to determine adjusted odds ratios (ORs).

The model for multivariate analysis was made choosing variables for the significance in the univariate comparison and for clinical relevance [13]

All statistics were performed using Statistical Package for Social Science (SPSS®) software version 22 (SPSS, Inc.).

### Ethics

The study conforms to the ethical guidelines of the 1975 Declaration of Helsinki (6th revision, 2008), as reflected in a priori approval by the institution’s human research committee at each participating study site. The study was approved by Ethic Committee of Fondazione Policlinico A. Gemelli IRCSS (ID 3076—protocol number: 0016460/21) as coordinator center.

### Outcome measures

The primary outcome of this study is the evaluation of the variation of seizure frequency in PwE within 90 days after the administration of the COVID-19 vaccine.

Secondary outcome measures included the assessment of the variables associated with seizure frequency worsening (i.e., demographic features, age at epilepsy onset, duration of epilepsy, epilepsy and seizure type, etiology, drug-resistant epilepsy, number of ASMs, ASM variation in the previous year, type of vaccine, variation of sleep habits, caffeine or alcohol intake).

## Results

### Description of the cohort

**Fig. 1:**
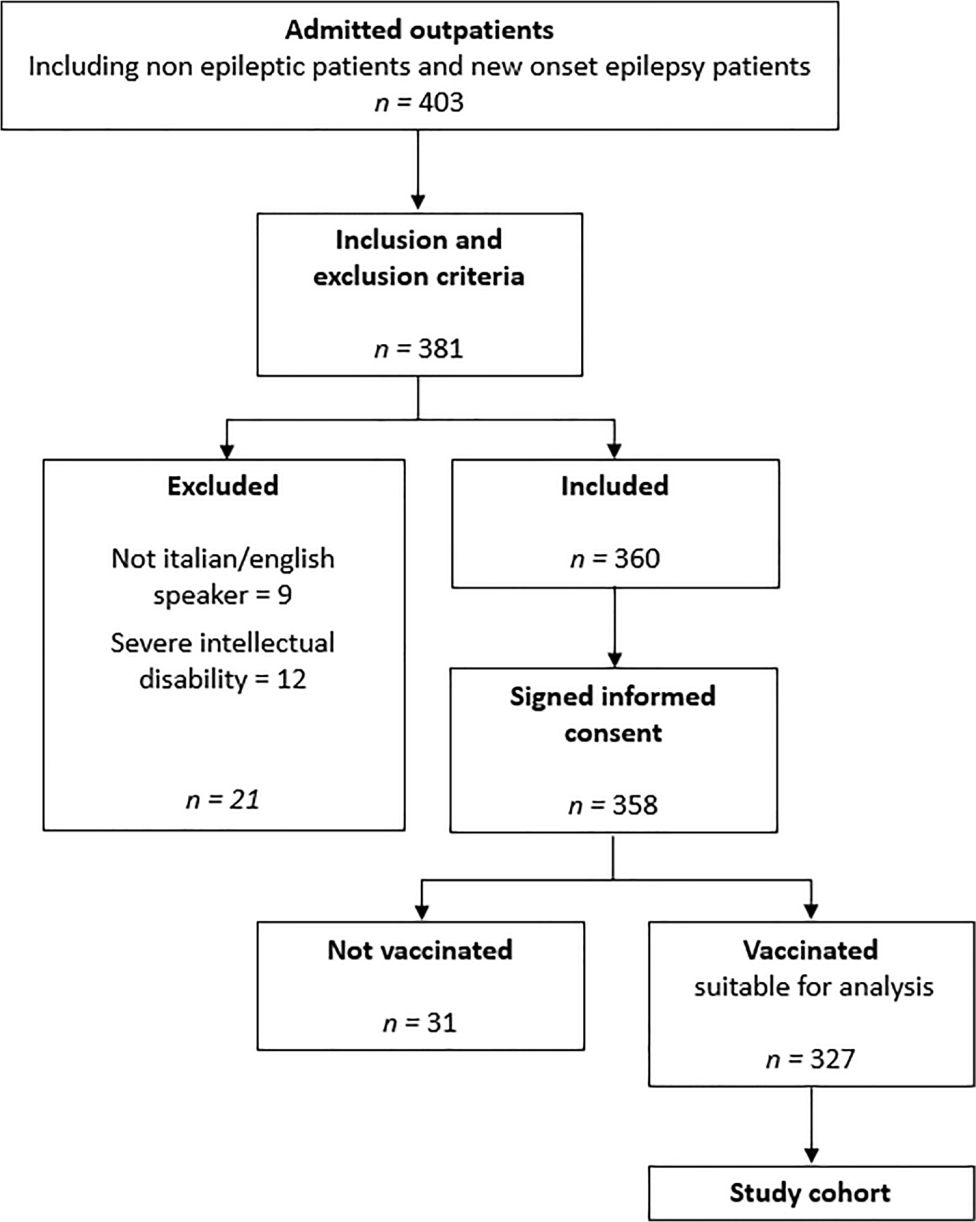
Flow-chart of the study depicting the enrolment process

In the period of study, a total of 403 patients were admitted to our outpatients’ clinics and screened for participation to the study. A total of 360 patients met the inclusion criteria and were asked to participate to the study (Fig. 1). The final total cohort consisted of 358 patients, 161 were males (45.0%) and 197 females (55.0%), with a mean age of 47.46 ± 19.04 (range 14–93). Focal seizure (283 patients, 79.1%), generalized epilepsy (73 patients, 20.4%), and unknown types of epilepsy (2 patients, 0.5%) were detected among participants. Seven patients (2%) presented a mild-to-moderate intellectual disability and needed a caregiver for providing accurate information about their clinical status. Of the overall cohort, 327 were vaccinated and 31 were not vaccinated.

Out of 358 patients, 229 patients were in monotherapy: 90 patients (25.14%) were treated with levetiracetam, 51 patients (14.25%) with sodium valproate, 39 patients (10.89%) with carbamazepine, 25 patients (6.98%) with lamotrigine, 9 patients (2.51%) with oxcarbazepine, 8 patients (2.23%) with phenobarbital, 5 patients (1.4%) with lacosamide, and 2 patients with topiramate (0.56%). One hundred fourteen patients (31.84%) took a combination of ≥ 2 ASMs. Fifteen patients did not take ASMs (4.19%) (Table 1).

**Table 1:** Demographic characteristics of the study cohort. *ASM*, antiseizure medication

**Demographic and clinical data**	Mean	SD	*n*	%
**Sex**				
Male			161	44.9
Female			197	55.1
**Age**	47.46	19.04		
**Vaccination choice**				
Vaccinated			327	91.3
Non-vaccinated			31	8.7
**Epilepsy duration (years)**	14.5	14.13		
**Monthly seizure frequency baseline**	1.59	7.38		
**Epilepsy type**				
Focal			283	79.1
Generalized			73	20.4
Unknown			2	0.5
**Epilepsy etiology**				
Structural			145	40.5
Infectious			4	1.1
Immune			5	1.4
Metabolic			1	0.3
Genetic			17	4.8
Unknown			185	51.8
**Drug resistance**				
No			300	83.8
Yes			58	16.2
**Seizure frequency pattern**				
< 1/year			245	69.0
> 1/year			48	13.5
> 1/month			20	5.6
> 1/week			25	7.0
> 1/day			17	4.8
**Number of ASMs**				
0			15	4,2
1			229	64
2			77	21,5
3			26	7,3
4			7	2
5			4	1,1
**ASMs variation in the previous year**				
No			53	15.9
Yes			281	84.1
**Changes in sleep habits**				
No			306	94.4
Yes			18	5.6

In the total cohort, a total of 14 patients had a previous COVID-19-documented infection.

The clinical and demographic characteristics of the total cohort are shown in Table 1.

### Vaccinated patients

All patients received vaccine doses according to the scheduled interval for each vaccine type.

A total of 257 patients received Pfizer/BNT162b2 (79.08%), 43 patients received the Moderna/mRNA1273 (13.23%), 24 patients the AstraZeneca/AZD122/ChAdOx1 n-CoV-19 (7.38%), and one patient the Janssen/Ad26 (0.31%).

Three hundred and two patients (92.35%) did not experience an increase in the seizure frequency (STABLE-group) whereas 25 patients (7.65%) had a seizure worsening (WORSE-group). Post-vaccine seizures occurred mainly in the 7 days following the administration of the vaccine (mean ± SD: 8.23 ± 8.19 days) and have never been associated with fever as well as with significant changes in sleep or alcohol intake habits. Patients in WORSE group did not experience any change in seizure type if compared to the pre-vaccine period. Only eight patients (2.45%) needed hospitalization for the recurrence of seizure after vaccination, but in the period examined, no cases of status epilepticus have been documented in our cohort. Breakthrough seizures (i.e., seizures occurring after at least 12 months of seizure freedom while on treatment) occurred in only two patients, in both cases after the second dose (Table 1).

When compared to STABLE-group, patients in the WORSE-group were treated with a mean higher number of ASMs (*p* = 0.003) and had a higher pre-vaccine seizure frequency (*p* < 0.001) (Table 2). Drug-resistant epilepsy was also associated with seizure worsening (*p* = 0.01) (Table 3). No differences were observed between the two groups concerning age, seizure and epilepsy type, etiology, and disease duration (Table 2).

**Table 2:** Univariate comparison between the subgroup of patients with seizure frequency worsening (WORSE) and the subgroup of patients with seizure stability (STABLE). *ASM*, antiseizure medication; *BDI*, Beck’s Depression Inventory

	**STABLE**	**WORSE**	**Vaccinated group**	**Mann–Whitney**	
	***n* = 302**	***n* = 25**	***n* = 327**		
	Mean ± SD	Mean ± SD	Mean ± SD	U-test	*p*
**Age**	48.05 ± 19.21	51.4 ± 18.78	48.31 ± 19.17	4147.0	0.413
**Epilepsy duration**	14.38 ± 14.22	15.9 ± 14.82	14.5 ± 14.25	4054.0	0.539
**Number of ASMs**	1.37 ± 0.8	2.0 ± 1.23	1.42 ± 0.85	4915.0	0.003
**Monthly seizure frequency baseline**	1.42 ± 6.93	5.60 ± 13.64	1.74 ± 7.70	5660.0	< 0.001
**Total sleep time**	8.04 ± 1.3	8.21 ± 1.1	8.05 ± 1.28	4037.0	0.245
**Sleep efficiency**	95.80 ± 5.51	95.22 ± 9.82	95.76 ± 5.92	3959.0	0.349
**BDI total Score**	2.6 ± 5.46	3.82 ± 6.02	2.69 ± 5.50	1808.0	0.605

**Table 3:** Univariate comparison between the subgroup of patients with seizure frequency worsening (WORSE) and the subgroup of patients with seizure stability (STABLE). *ASM* antiseizure medication

		**STABLE**		**WORSE**		**Vaccinated group**	
		***n* = 302**		***n* = 25**		***n* = 327**	${{\varvec{\chi}}}^{2}$
		***n***	**(%)**	***n***	**(%)**	***n***	***p***
**Sex**							0.837
Male		137	91.95	12	8.05	149	
Female		165	92.7	13	7.3	178	
**Vaccine type**							0.988
Pfizer/BNT162b2		237	92.22	20	7.78	257	
Moderna/mRNA1273		40	93.02	3	6.98	43	
AstraZeneca/AZD122/ChAdOx1 n-CoV-19		22	91.67	2	8.33	24	
Janssen/Ad26		1	100.0	0	0.0	1	
**Epilepsy type**							0.188
Focal		238	91.19	23	8.81	261	
Generalized		62	96.88	2	3.13	64	
Unknown		0	0	0	0	0	
**Epilepsy etiology**							0.382
Structural		126	94.03	8	5.97	134	
Infectious		3	75.0	1	25.0	4	
Immune		4	80.0	1	20.0	5	
Metabolic		1	100.0	0	0.0	1	
Genetic		17	100.0	0	0.0	17	
Unknown		150	90.91	15	9.09	165	
**Drug resistance**							0.01
No		265	94.64	15	5.36	280	
Yes		37	78.72	10	21.28	47	
**Seizure frequency pattern**							< 0.001
< 1/year		225	98.68	3	1.32	228	
> 1/year		33	82.5	7	17.5	40	
> 1/month		15	75	5	25	20	
> 1/week		17	73.91	6	26.09	23	
> 1/day		12	75	4	25	16	
**ASM variation/previous year**							0.087
No		46	86.79	7	13.21	53	
Yes		255	82.72	17	6.25	272	
**Hospital access**							0.123
No		289	92.63	23	7.37	312	
Yes		6	75.0	2	25.0	8	
**Changes in sleep habits**							0.159
No		278	92.67	22	7.33	300	
Yes		15	83.33	3	16.67	18	

Patients in the WORSE-group had also a more severe seizure frequency pattern (*p* < 0.001) (Table 3).

All the comparisons are summarized in Table 2 and Table 3.

**Fig. 2:**
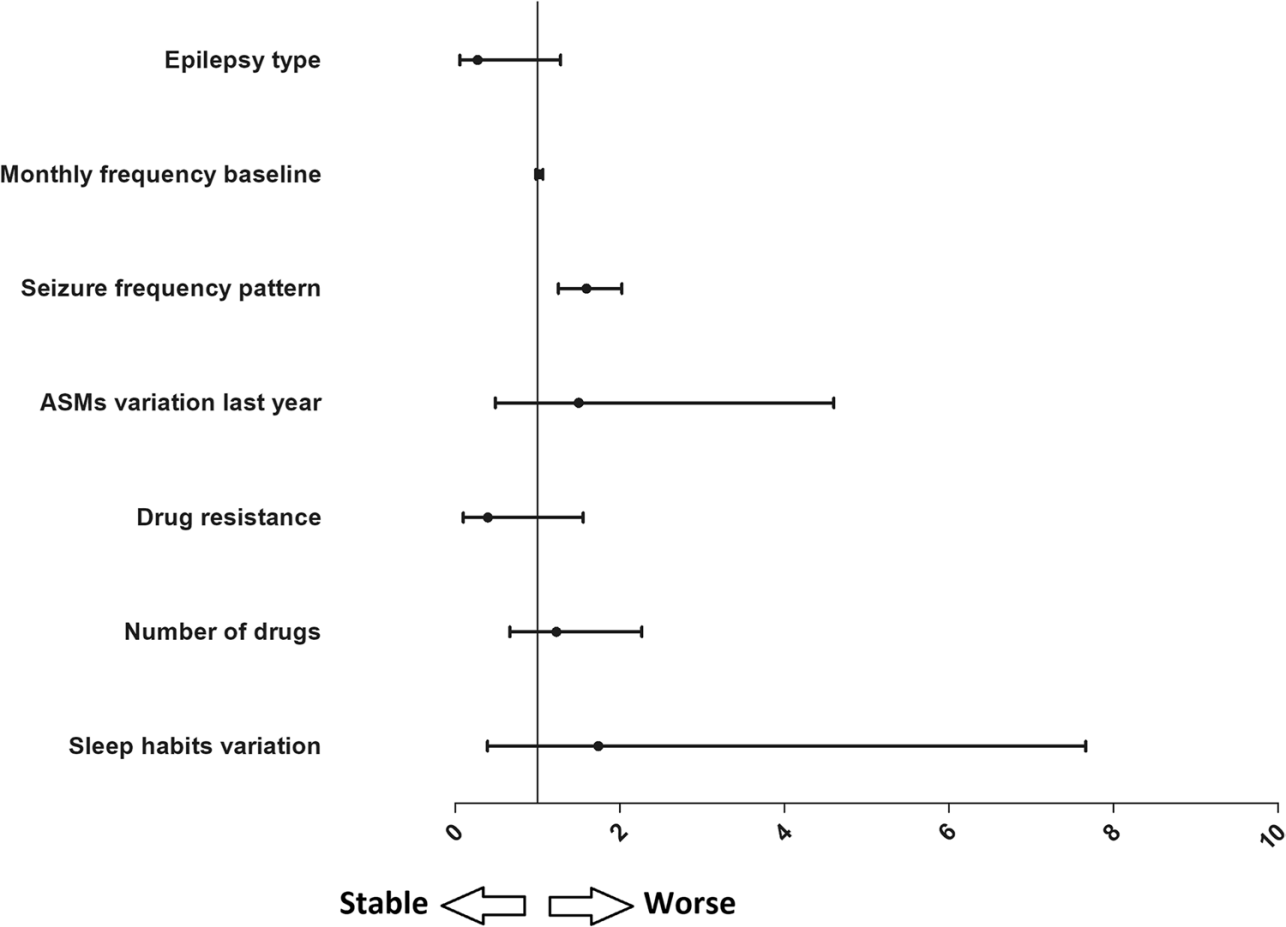
Multivariate analysis with seizure worsening as dependent variable

Multivariate analysis of the vaccinated group showed that only the frequency pattern (confidence interval [CI] = 1.257–2.028; *p* < 0.001) was significantly associated with seizure worsening (Fig. 2).

### Non-vaccinated patients

The non-vaccinated patients represented a small percentage of the total cohort. The causes of non-vaccination or vaccination hesitancy were mostly fear of aggravating epilepsy, pregnancy, or personal reasons (i.e., “anti-vaxxer”).

### Vaccine side effects

Sixty-six patients (20.18%) experienced at least one side effect after the first dose of vaccination, and 70 patients (21.41%) had at least one side effect after the second dose. The side effects were all mild and fleeting and included local skin reactions (e.g., itchiness, rash, and swelling) and muscular pain and minor systemic side effects such as flu-like syndrome, fever, myalgia, arthralgia, fatigue, and headache. Thirty-one patients experienced fever after vaccination (7 after the first administration and 24 after the second dose). None of the aforementioned conditions required hospitalization.

## Discussion

This multicentric observational study aimed to explore the safety and tolerability profile of COVID-19 vaccination in the short term in a large Italian cohort of PwE.

Only a small percentage of PwE in our cohort (7.65%) experienced seizure worsening. These patients were affected mostly by a poor-controlled epilepsy, and/or a drug-resistant epilepsy, and were on ASMs polytherapy before vaccination.

The duration of epilepsy as well as the age of the patients, on the other hand, did not affect the worsening of seizures. In our cohort, there were no cases of status epilepticus and the hospitalization for seizure worsening occurred only in eight patients.

A recent Chinese multicentric study on 491 PwE demonstrated that fewer than 10% of the patients reported the occurrence of seizure after COVID-19 vaccination [14]. In the aforementioned study, the change of seizure frequency was assessed considering the period from the first injection of vaccine to 1 week afterward [14]. Massoud et al. assessed the side effects of 82 PwE who received Pfizer/BNT162b2 and ChAdOx1nCoV-19 vaccination and, similarly to our data, most patients (93.9%) did not report seizure worsening after vaccination. One patient presented status epilepticus [15]. In a survey on 27 patients, one patient reported increased seizure frequency 1 day after the first COVID-19 vaccination was administered, and one reported the occurrence of a new seizure type [16].

The correlation between any vaccines and seizures, epilepsy, and/or epileptic encephalopathies has been comprehensively studied, in particular in pediatric population [8]. Diphtheria and tetanus toxoids and whole-cell pertussis (DTP) vaccine and measles, mumps, and rubella (MMR) vaccine are associated in children with a significant risk of febrile seizures on the day of receipt of DTP vaccine and 8 to 14 days after MMR vaccine. Anyway, these risks were not associated with long-term consequences [6]. Studies on the adult population are less represented. Inactivated vaccines such as inactivated influenza vaccines were associated with very low increased risk of seizures in patients with epilepsy with a mean age at vaccination of 41 (1.01, 95% confidence interval 0.74 to 1.39) after 1 week post-vaccination but not a long-term increase [17].

Vaccines have not been related with afebrile seizures, and several studies and reviews concluded that immunizations do not trigger the onset of epilepsy [8]. In our cohort, the patients who experienced seizure worsening after vaccination did not have concomitant fever. This finding may question a direct effect of vaccination, even if these patients were affected by a more severe and less controlled epilepsy. Indeed, our multivariate analysis confirms that the only factor associated with seizure frequency worsening was the pre-vaccine seizure frequency pattern. Therefore, it is possible to hypothesize that vaccination does not have a direct causative effect in the modification of the seizure frequency. As a matter of fact, the patients who worsened were affected by a more severe and less controlled epilepsy.

Regarding general side effects, several studies concluded that their occurrence is comparable between general population and PwE [15, 16].

In our cohort, vaccine general side effects were mild and transient and occurred in approximately 20% of patients both after the first and second dose. These data were consistent with those reported in the general population [18]. These side effects were equally distributed among all vaccines.

Vaccine hesitancy in PwE still remains a global issue, being the fear of aggravating epilepsy the major concern [19].

There are no known interactions between the ASMs and COVID-19 vaccines [20].

The main strength of the study is the number of patients recruited and the multicenter design, which allow minimizing the effects of possible bias of selection. Another strength is the characteristic of the study cohort, which is highly representative of the population of patients with epilepsy.

A limitation of the study is the self-reporting of seizures and side effect and the exclusion of patients with cognitive impairment and intellectual disability when a caregiver was not available.

## Conclusion

The present study demonstrates that COVID-19 vaccines have a good safety and tolerability profile in the short term in PwE, with a minor impact on seizure worsening.

Given the extent of COVID-19 spread all over the world, it is essential to develop large-scale immunity in an effort to stop the pandemic. In our cohort of COVID-19-vaccinated epileptic patients, only a little percentage had a transient short-term increase in seizure frequency, with minimal impact on the healthcare system.

Uncontrolled epilepsy was the most important predictive factor of seizure recurrence after COVID-19 vaccination. Vaccination per se does not represent a major risk of aggravating epilepsy with benefits outweighing risks.
